# Supplementary material for: Prebiotic Combinations Effects on the Colonization of Staphylococcal Skin Strains
Source: Microorganisms. 2020 Dec 24;9(1):37. doi: 10.3390/microorganisms9010037 (PMC7824076; doi:10.3390/microorganisms9010037)
Supplement: Supplementary file 1 [file microorganisms-09-00037-s001.pdf]

Supplementary information

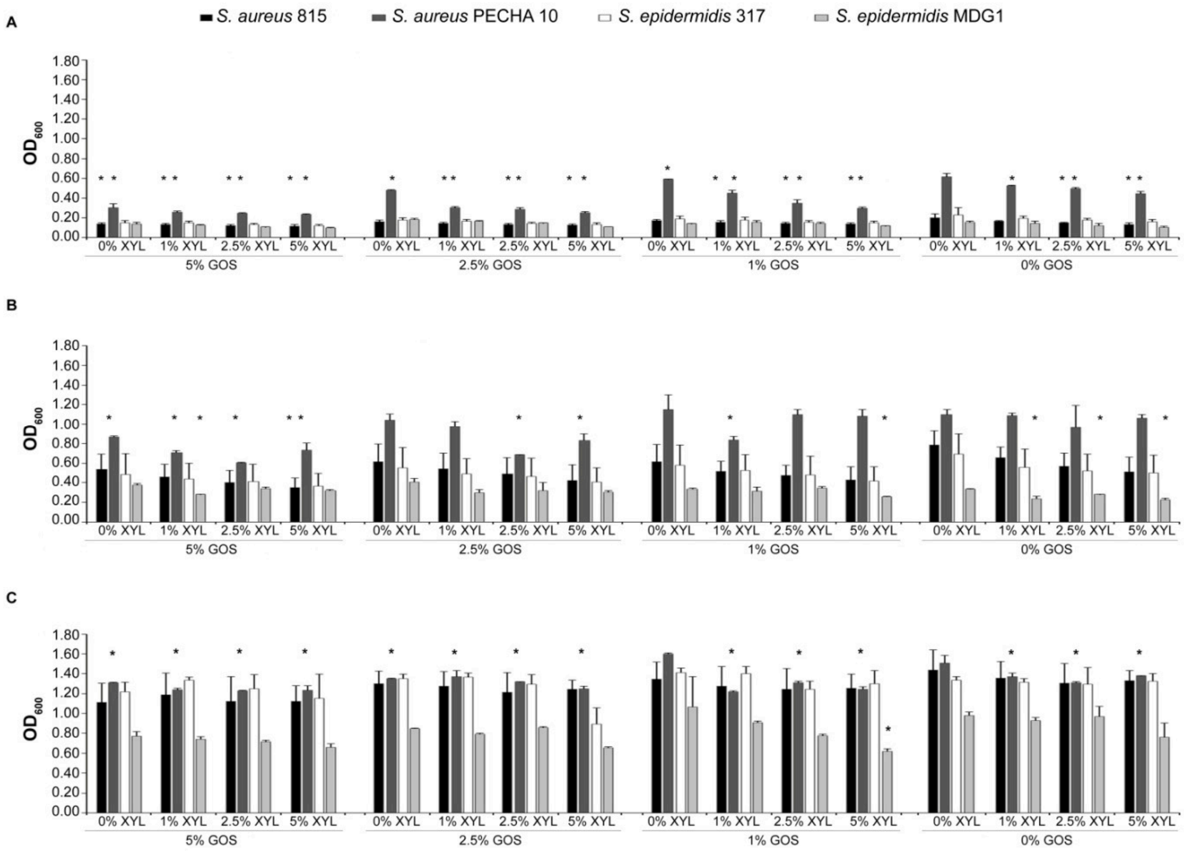

Figure S1. Optical density (OD_{600}) of *S. aureus* 815, *S. aureus* PECHA 10, *S. epidermidis* 317 and *S. epidermidis* MDG1 planktonic growth after 3 h (**A**), 6 h (**B**), 24 h (**C**) of contact with Xylitol (XYL) and Galacto-OligoSaccharide (GOS) at different concentrations (1, 2.5, 5%). *statistically significant in respect to the control ($p < 0.05$).

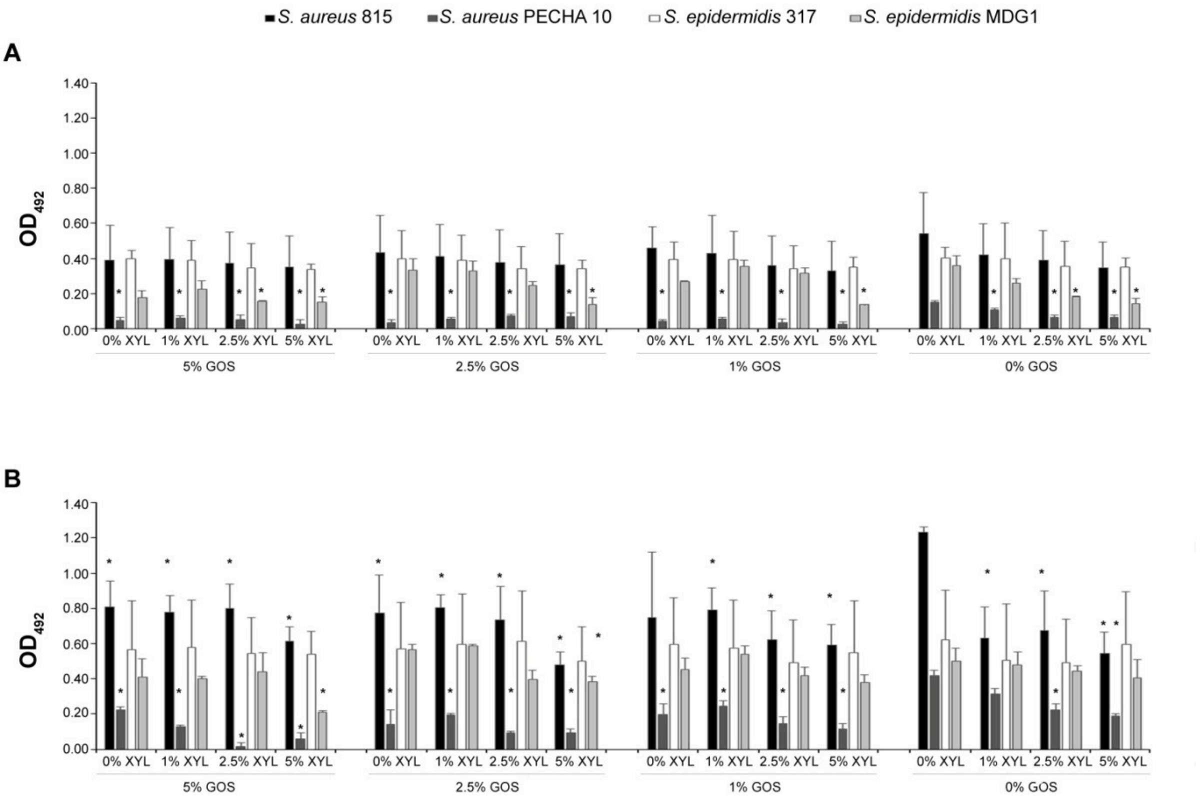

Figure S2. Biomass quantification (OD_{492}) of *S. aureus* 815, *S. aureus* PECHA 10, *S. epidermidis* 317 and *S. epidermidis* MDG1 biofilm formation after treatment with different concentrations of Xylitol (XYL) and Galacto-OligoSaccharide (GOS) at 3 h (**A**) and 24 h (**B**), respectively. *statistically significant in respect to the control ($p < 0.05$).

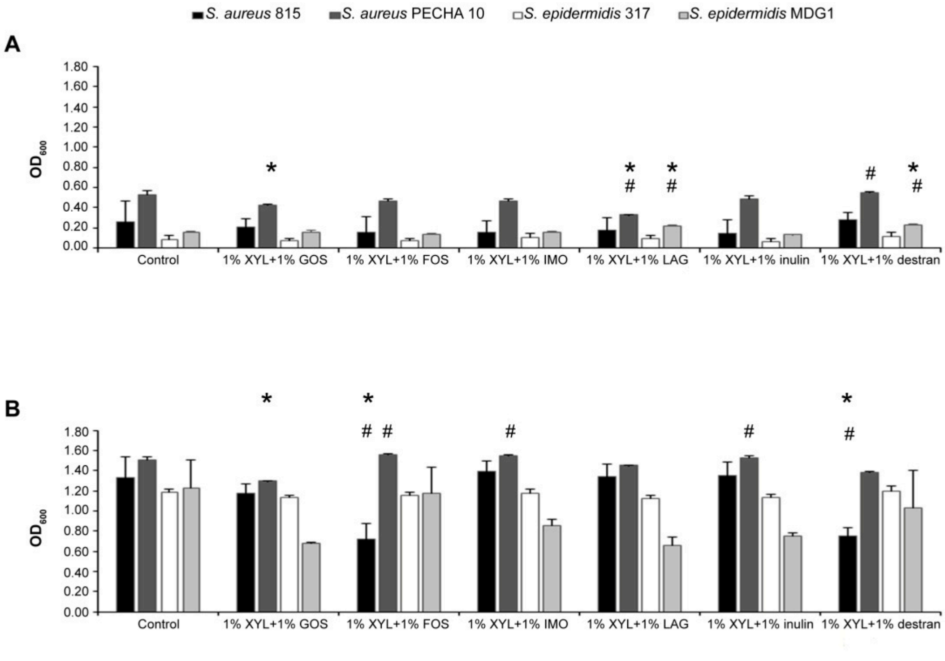

Figure S3. Optical density (OD_{600}) of *S. aureus* 815, *S. aureus* PECHA 10, *S. epidermidis* 317 and *S. epidermidis* MDG1 planktonic growth after 3 h (**A**), 24 h (**B**) of contact with 1% of Xylitol (XYL) and 1% of skin emerging prebiotics: FOS or IMO or LAG or inulin or dextran. *statistically significant in respect to the control ($p < 0.05$).[#]statistically significant in respect to the RC (1% XYL + 1% GOS)($p < 0.05$). For abbreviations, see materials and methods.

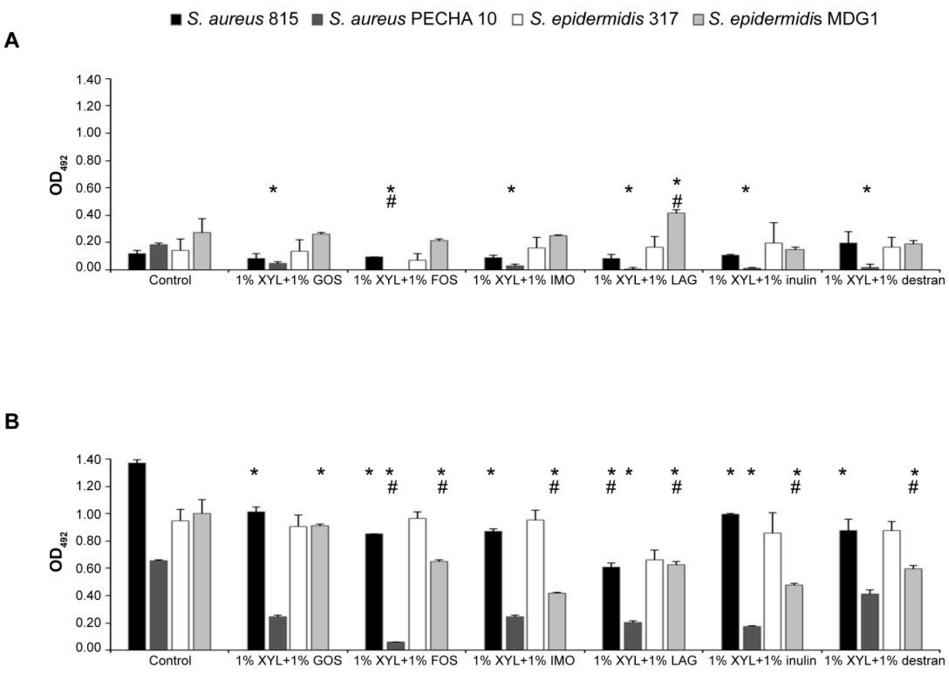

Figure S4. Biomass quantification (OD492) of *S. aureus* 815, *S. aureus* PECHA 10, *S. epidermidis* 317 and *S. epidermidis* MDG1 biofilm formation after treatment with 1% XYL and 1% FOS or IMO or LAG or inulin or dextran at 3 h (**A**) and 24 h (**B**), respectively. *statistically significant in respect to the control ($p < 0.05$).#statistically significant in respect to the RC (1% XYL + 1% GOS)($p < 0.05$). For abbreviations, see materials and methods.
